# Supplementary material for: Identification of photoperiod-induced specific miRNAs in the adrenal glands of Sunite sheep (Ovis aries)
Source: Front Vet Sci. 2022 Jul 22;9:888207. doi: 10.3389/fvets.2022.888207 (PMC9354845; doi:10.3389/fvets.2022.888207)
Supplement: Supplementary file 1 [file Data_Sheet_1.ZIP › Supplementary file/miRDeep2 file.zip!/miRDeep2 file/Novel_Structure/SPLP42A1/Novel_73.pdf]

Provisional ID	:	NC_019463.2_12260
Score total	:	70.5
Score for star read(s)	:	3.9
Score for read counts	:	63.3
Score for mfe	:	1.7
Score for randfold	:	1.6
Score for cons. seed	:	
Total read count	:	136
Mature read count	:	135
Loop read count	:	0
Star read count	:	1

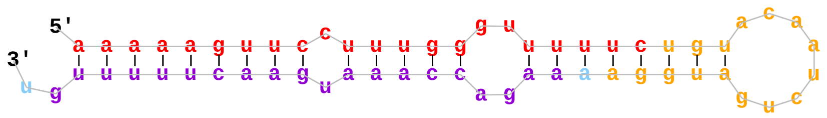

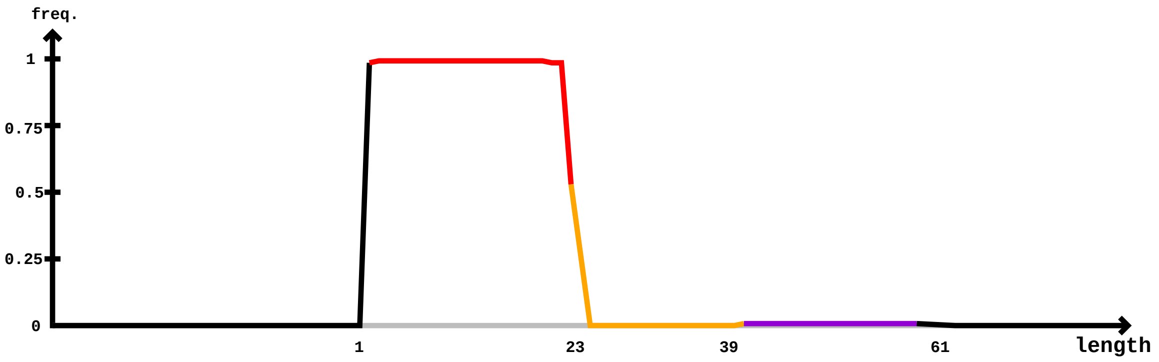

Mature Star

```
5'- auaucuauuucaaguaucuauuggguuggacaaaaaguuccuuuggguuuuucuguacaaucugauggaaaagaccaaaugaacuuuuuguuaaauccaauauuuauuca  -3'   obs
    auaucuauuucaaguaucuauuggguuggacaaaaaguuccuuuggguuuuucuguacaaucugauggaaaagaccaaaugaacuuuuuguuaaauccaauauuuauuca        exp
    ............((((..(((((((((.((((((((((((.(((((..((((((((........))))))))..))))).)))))))))))).)))))))))..))))..   reads   mm   sample
    ...............................aaaaaguuccuuuggguuu............................................................   1       0    seq
    ...............................aaaaaguucGuuuggguuuuu..........................................................   62      1    seq
    ...............................aaaaaguucGuuuggguuuuuc.........................................................   66      1    seq
    ...............................aaaaaguucAuuuggguuuuucu........................................................   1       1    seq
    ...............................aaaaaguucGuuuggguuuuucu........................................................   4       1    seq
    ................................aaaaguucAuuuggguuuuucu........................................................   1       1    seq
    ......................................................................aagaccaaaugaacuuuuug....................   1       0    seq
```
